# Position and orientation inference via on-board triangulation

**DOI:** 10.1371/journal.pone.0180089

**Published:** 2017-06-23

**Authors:** Madhu Advani, Daniel S. Weile

**Affiliations:** 1 Center for Brain Science, Harvard University, Cambridge, Massachusetts, United States of America; 2 Department of Electrical and Computer Engineering, University of Delaware, Newark, Delaware, United States of America; Tongji University, CHINA

## Abstract

This work proposes a new approach to determine the spatial location and orientation of an object using measurements performed on the object itself. The on-board triangulation algorithm we outline could be implemented in lieu of, or in addition to, well-known alternatives such as Global Positioning System (GPS) or standard triangulation, since both of these correspond to significantly different geometric pictures and necessitate different hardware and algorithms. We motivate the theory by describing situations in which on-board triangulation would be useful and even preferable to standard methods. The on-board triangulation algorithm we outline involves utilizing *dumb* beacons which broadcast omnidirectional single frequency radio waves, and *smart* antenna arrays on the object itself to infer the direction of the beacon signals, which may be used for onboard calculation of the position and orientation of the object. Numerical examples demonstrate the utility of the method and its noise tolerance.

## Introduction

Technology for tracking and locating objects has been revolutionized by the global positioning system (GPS) [1]. From consumer applications like navigation guidance to military applications tracking missiles and aircrafts, GPS has proven to have a variety of uses. However, complete reliance on GPS may be a risky or suboptimal strategy in situations where greater accuracy is desired or in the scenario that GPS systems go offline or are blocked. For instance, GPS signals can be blocked by mountain ranges or buildings, a fact which has already motivated the use of WiFi to triangulate electronic devices in buildings [2].

Terrestrial triangulation is an alternative method for inferring the location of an object using estimates of the angles from various known reference points to the object being tracked. Even with growing reliance on GPS technology, triangulation has various applications from low cost environmental monitoring [3] to vehicular detection of pedestrians [4]. More recently, triangulation has been applied to collections of collaborating robots engaged in formation control or collision avoidance. While both range and angle sensors may be included in such systems, angular information can be more reliable, for instance when utilizing on-board cameras [5, 6]. Partly for this reason, recent works [6–8] have applied iterative numerical algorithms based solely on angle measurements to locate objects in some general reference frame. These algorithms rely on iteratively broadcasting angle estimates (observing relative angles between robots) to update estimates of angles in a global reference frame. Once these angles are known, and assuming knowledge of several reference locations, something similar to standard triangulation can be performed between robots to estimate positions.

However, there are two potential drawbacks to using standard triangulation which we mention here. First, in practice it often requires setting up specialized equipment (e.g. triangulation towers or smart antenna arrays) to perform angle estimates within the line of sight of the object, which may not always be logistically feasible. Second, the calculations are not done in an on-board manner, so that the location information must still be transmitted to the tracked object. This second missing feature of triangulation would be very useful, for instance, in a smart vehicle or self-correcting rocket which had the capacity to perform on-board computations. To ameliorate the aforementioned drawbacks of standard triangulation, we propose a system for on-board triangulation and derive an algorithm that can be performed entirely within the processor of the tracked object. Because the heart of the system resides on the tracked object, it requires only limited hardware externally.

On-board triangulation requires the tracked object to detect the angle to a set of reference points at known positions, which we will refer to as *beacons*. While our algorithm could potentially be used with several different technologies, the scenario we envisage here is a collection of *dumb* beacons, which would each broadcast electromagnetic waves at particular frequencies. The advantage of using such dumb beacons is that they would be cheaper and more portable than, for instance, triangulation towers. The tracked object then requires an on-board microprocessor to perform calculations, and a smart antenna array to accurately infer the direction of incoming signal relative to that of the array. The antenna array uses the phase difference in the incident signals at different antennas in the array to infer the signal direction [9] and can compute the direction of the multiple beacons, assuming they are broadcasting at different frequencies or are otherwise individually identifiable.

Of course, all angle measurements are made in the frame of the tracked object, which will, in general, be rotated relative to the ground frame. This mismatch between reference frames introduces additional difficulties not present in standard triangulation. To better understand this, note that it is not even obvious how many beacons are needed to perform on-board triangulation. More beacons are clearly required for the on-board method than for standard triangulation for the following reason: In the case of standard triangulation in two dimensions, only two angles (based on directions from reference points to the tracked object) are needed. When this information is combined with the side length linking the two reference points, the result is a unique triangle (due to the side-angle-side theorem of Euclidean geometry) and thus a unique object location. Unfortunately, in the case of on-board triangulation in two dimensions, two beacons will give you only one angle and the opposing side the triangle, which is not enough information to uniquely define the triangle. In this paper, we will demonstrate that three beacons are generally sufficient to perform on-board tracking in two dimensions. In some cases, however, three beacons lead to multiple predicted locations so that a fourth beacon or some additional information, such as history of the position, may be required.

We also note that the method of inferring angles to beacons is very different from the technology used by GPS, which instead estimates the distances signals travel from multiple satellites, based on time stamps and precisely timed clocks. The GPS approach of utilizing distances rather than angles to determine location is known as *trilateration*. To solve the problem of determining location via on-board triangulation, we propose a new algorithm, which at its heart is rooted in well-known Euclidean geometric theorems, and is entirely analytic in its implementation.

Finally, while there are some similarities between the problem of locating an object in space described here and that of determining the reliability of ad-hoc wireless networks [10], the design of the antennas employed will be very different. Directive antennas in communications networks ensure high fidelity transmissions, but nulls in the beacon antenna patterns would leave the location system described here blind in some directions. Of course, the impossibility creating an isotropic antenna means that the beacons will necessarily radiate in some directions more than others (at least in three dimensions), but so long as the object being located receives a signal from all of the beacons, the algorithm presented here will work. Moreover, as will be shown below, with some information about the location of the object, some types of directivity will make the location algorithm more efficient.

The algorithm we describe in this letter applies to on-board triangulation in two dimensions; limiting the discussion to 2D permits a more terse and intuitive description of the method. With some additional technical complexity the same idea can be extended to higher dimensional space, but we leave this as a direction for future work. The next section will therefore describe the algorithm in detail. We then demonstrate the algorithm on simulated data, and provide discussion and suggestions for future extensions of this method.

## Algorithm

The underlying on-board triangulation algorithm can be described in a paragraph: Begin by choosing a pair of beacons and computing the angle between them from the point of view of the object. Next, use this angle to construct a *plausible point*, i.e., a point in space from which the object would perceive the same angle between vectors directed at the two beacons. This plausible point will not generally be the true location of the object, but is nevertheless useful because it allows one to construct a *plausible curve* that includes all points in space from which the object would perceive the same angle, and thus must include the true object position. These curves are in fact circular arcs according to the inscribed angle theorem of Euclid. We then repeat this process with susequent pairs of beacons until a unique intersection of the plausibility curves exist; this intersection is the inferred object location.

### Constructing plausible points

Imagine a set of *N* beacons located at positions **b**^1^, **b**^2^, …, **b**^*N*^ located in a two-dimensional space. Choose a pair of beacon locations **b**^*α*^ and **b**^*β*^ for some *α*, *β* ∈ {1, …*N*}. Since the tracked object infers the direction of each signal, it is also capable of calculating the angle between pairs of incoming signals. Call this angle *θ*_*αβ*_.

Given *θ*_*αβ*_, a straightforward method to find a plausible point ${\tilde{s}}^{\alpha \beta }$ is to start at the midpoint of the line segment connecting **b**^*α*^ and **b**^*β*^ and then to follow the perpendicular bisector of the line joining these two beacons to a distance *l*_*αβ*_ at which the angle is between the two beacons becomes *θ*_*αβ*_. Thus, *l*_*αβ*_ satisfies

$$\begin{array}{c} l_{\alpha \beta }=\frac{∥b^{\beta }-b^{\alpha }∥}{2\text{tan}(\frac{\theta_{\alpha \beta }}{2})}, \end{array}$$ (1)

where ∥ ⋅ ∥ denotes the Euclidean norm. In turn, this yields two plausible points:

$$\begin{array}{c} {\tilde{s}}^{\alpha \beta }=\frac{1}{2}(b^{\alpha }+b^{\beta })\pm l_{\alpha \beta }\frac{{\left(b^{\beta }-b^{\alpha }\right)}^{⊥}}{∥b^{\beta }-b^{\alpha }∥}. \end{array}$$ (2)

In this equation, the notation **u**^⊥^ represents the (counter-clockwise) perpendicular transformation of the vector **u** = (*u_x_*, *u_y_*) to (−*u*_*y*_, *u*_*x*_). There are two different points produced by the equation because two possible directions can be chosen for a ray along the perpendicular to a given line segment in a plane. We can now use either of these two plausible points to construct a curve with the property that from the perspective of each point on the curve, the angle between the two beacons *θ*_*αβ*_ remains fixed. Constructing such curves using a plausible point is discussed in the next section.

### Constructing plausible curves

In this section, we explain how to construct a plausible curve of positions from which the same relative angle *θ*_*αβ*_ between the signal from beacon locations **b**^*α*^ and **b**^*β*^ is maintained. To construct plausible curves we use the inscribed angle theorem (see e.g. [11]). Given three points *A*, *B*, and *C* on the circumference of a circle centered at *O*, the theorem states ∠*ABC* = 2∠*AOC*. Conversely, the curve created by moving the point *B* to preserve ∠*ABC* = *θ*_*αβ*_ given fixed points *A* and *C* is a circle.

To actually construct such this circle, we note that it is possible to construct a circle that intersects any three points which are not collinear. There are several approaches to this problem, all of which share the feature that they are simple in theory but tedious in execution. For brevity we simply state the solution here [12]. The co-ordinates of the center **c** = (*c_x_*, *c_y_*) of the circle containing **b**^*α*^, **b**^*β*^, and ${\tilde{s}}^{\alpha \beta }$ are given by

$$\begin{array}{c} c_{x}=-\frac{d}{2a},\;\;c_{y}=-\frac{e}{2a}, \end{array}$$ (3)

and its radius is given by

$$\begin{array}{c} r=\frac{\sqrt{d^{2}+e^{2}-4af}}{2a}, \end{array}$$ (4)

and the variables in these equations are defined by the determinants

$$\begin{array}{c} a=|\begin{array}{ccc} b_{x}^{\alpha } & b_{y}^{\alpha } & 1\\ b_{x}^{\beta } & b_{y}^{\beta } & 1\\ {\tilde{s}}_{x}^{\alpha \beta } & {\tilde{s}}_{y}^{\alpha \beta } & 1 \end{array}|,\;d=-|\begin{array}{ccc} {\left(b_{x}^{\alpha }\right)}^{2}+{\left(b_{y}^{\alpha }\right)}^{2} & b_{y}^{\alpha } & 1\\ {\left(b_{x}^{\beta }\right)}^{2}+{\left(b_{y}^{\beta }\right)}^{2} & b_{y}^{\beta } & 1\\ {\left({\tilde{s}}_{x}^{\alpha \beta }\right)}^{2}+{\left({\tilde{s}}_{y}^{\alpha \beta }\right)}^{2} & {\tilde{s}}_{y}^{\alpha \beta } & 1 \end{array}|,\\ e=|\begin{array}{ccc} {\left(b_{x}^{\alpha }\right)}^{2}+{\left(b_{y}^{\alpha }\right)}^{2} & b_{x}^{\alpha } & 1\\ {\left(b_{x}^{\beta }\right)}^{2}+{\left(b_{y}^{\beta }\right)}^{2} & b_{x}^{\beta } & 1\\ {\left({\tilde{s}}_{x}^{\alpha \beta }\right)}^{2}+{\left({\tilde{s}}_{y}^{\alpha \beta }\right)}^{2} & {\tilde{s}}_{x}^{\alpha \beta } & 1 \end{array}|,\;f=-|\begin{array}{ccc} {\left(b_{x}^{\alpha }\right)}^{2}+{\left(b_{y}^{\alpha }\right)}^{2} & b_{x}^{\alpha } & b_{y}^{\alpha }\\ {\left(b_{x}^{\beta }\right)}^{2}+{\left(b_{y}^{\beta }\right)}^{2} & b_{x}^{\beta } & b_{y}^{\beta }\\ {\left({\tilde{s}}_{x}^{\alpha \beta }\right)}^{2}+{\left({\tilde{s}}_{y}^{\alpha \beta }\right)}^{2} & {\tilde{s}}_{x}^{\alpha \beta } & {\tilde{s}}_{y}^{\alpha \beta } \end{array}|.\;\; \end{array}$$ (5)

### Intersections of plausible curves

**Fig 1:**
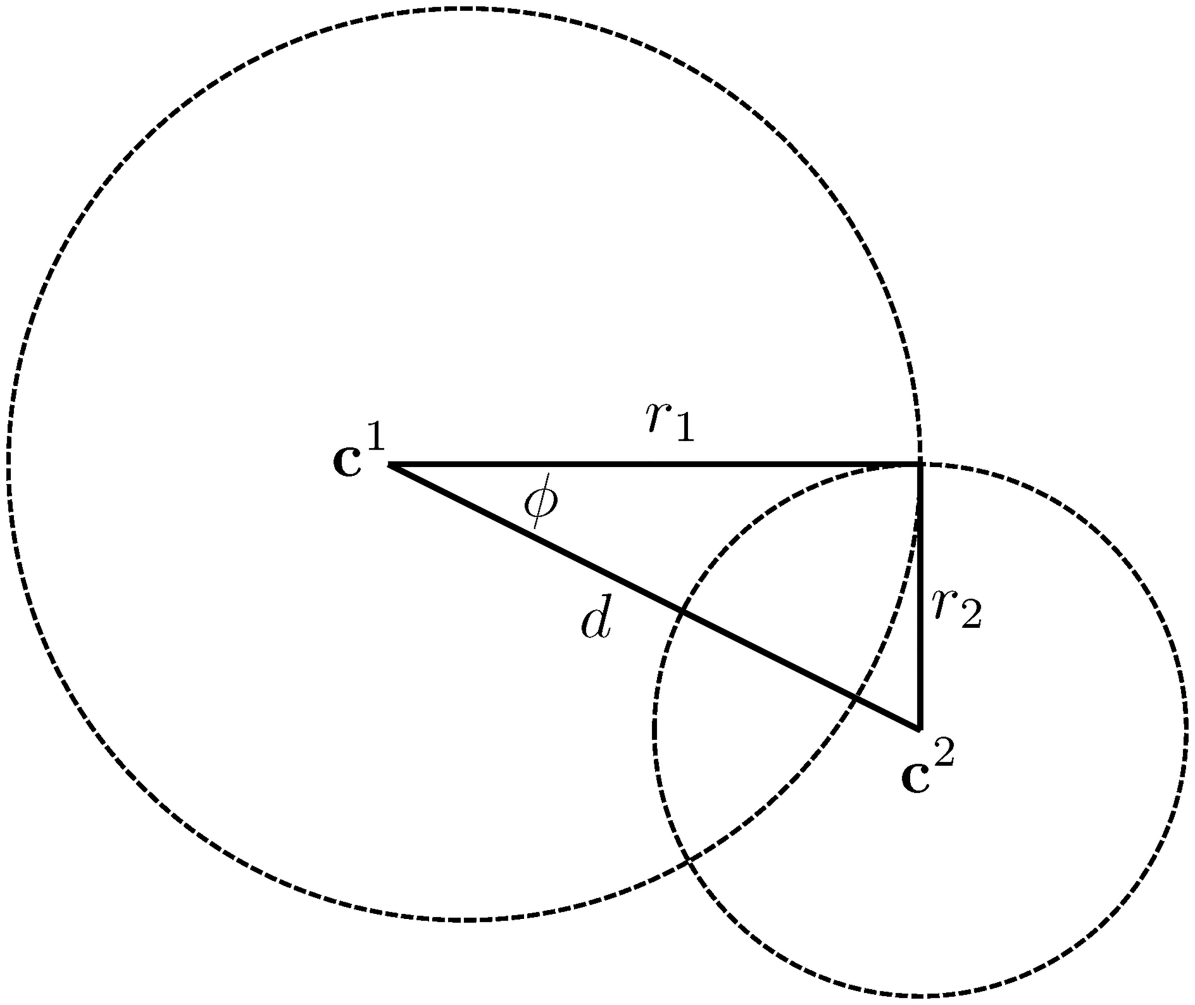
Determining the intersection points of a pair of circles.

Given more than two beacons, we can repeat the process of the previous section for each pair. A feasible location of the object will be at the intersection of a pair of circles (Fig 1). Begin by considering two circles with centers $\left(c_{x}^{1},c_{y}^{1}\right)$ and $\left(c_{x}^{2},c_{y}^{2}\right)$ and radii *r*_1_ and *r*_2_ respectively. Letting *d* = ∥**c**^1^ − **c**^2^∥, define *ϕ* as the angle measured counterclockwise from the line between the center of the two circles. This angle can be computed with the law of cosines:

$$\begin{array}{c} r_{1}^{2}+d^{2}-2r_{1}d\text{cos}\phi =r_{2}^{2}. \end{array}$$ (6)

Rearranging the previous equation to determine the angle of the intersection point yields

$$\begin{array}{c} \text{cos}\phi =\frac{r_{1}^{2}+d^{2}-r_{2}^{2}}{2r_{1}d}. \end{array}$$ (7)

Of course, two circles may be separated or lie within one another and thus need not always intersect. It follows from the law of cosines that there will only be a non-empty set of intersection points if

$$\begin{array}{c} |\frac{r_{1}^{2}+d^{2}-r_{2}^{2}}{2r_{1}d}|=\left|\text{cos}\phi \right|\leq 1. \end{array}$$ (8)

In the current application, circles can only avoid intersection if the wrong sign is chosen for some feasible ${\tilde{s}}^{\alpha \beta }$, and if more than three beacons is used. In any case, nonintersection is a boon: It can be used to eliminate a circle from consideration. To find the location of the intersection points **s**^*I*^, define a unit vector in the direction between the centers of the two circles:

$$\begin{array}{c} \hat{u}=\frac{\left(c_{2}-c_{1}\right)}{∥c_{2}-c_{1}∥}. \end{array}$$ (9)

The intersection points are then given by

$$\begin{array}{c} s^{I}=c_{1}+r_{1}(\text{cos}\phi \hat{u}\pm \text{sin}\phi {\hat{u}}^{⊥}). \end{array}$$ (10)

Although this results in multiple intersection points, we note that infeasible intersection points can be discarded easily: if the pair of beacons chosen to construct each of the plausible curves have a beacon in common with each other, this beacon will show up as an intersection point, which we can discount as a feasible point for the object. Even this observation is not actually needed in the case of three beacons since we will search for the set of intersection point of the three circles generated using each beacon pair, each of which excludes one beacon. Additionally, a feasible intersection point is constrained to lie on the same side of the circular arc between A and B as the plausible point. Finally, determining the orientation of the object follows immediately once its position **s** is determined: the angle of arrival of a signal measured from beacon **b**_*i*_ is simply the object’s orientation relative to the direction of the signal in a general reference frame given by (**s** − **b**_*i*_). Thus, adding the angle of this beacon-to-object direction to the detected angle yields the desired orientation.

## Simulations

**Fig 2:**
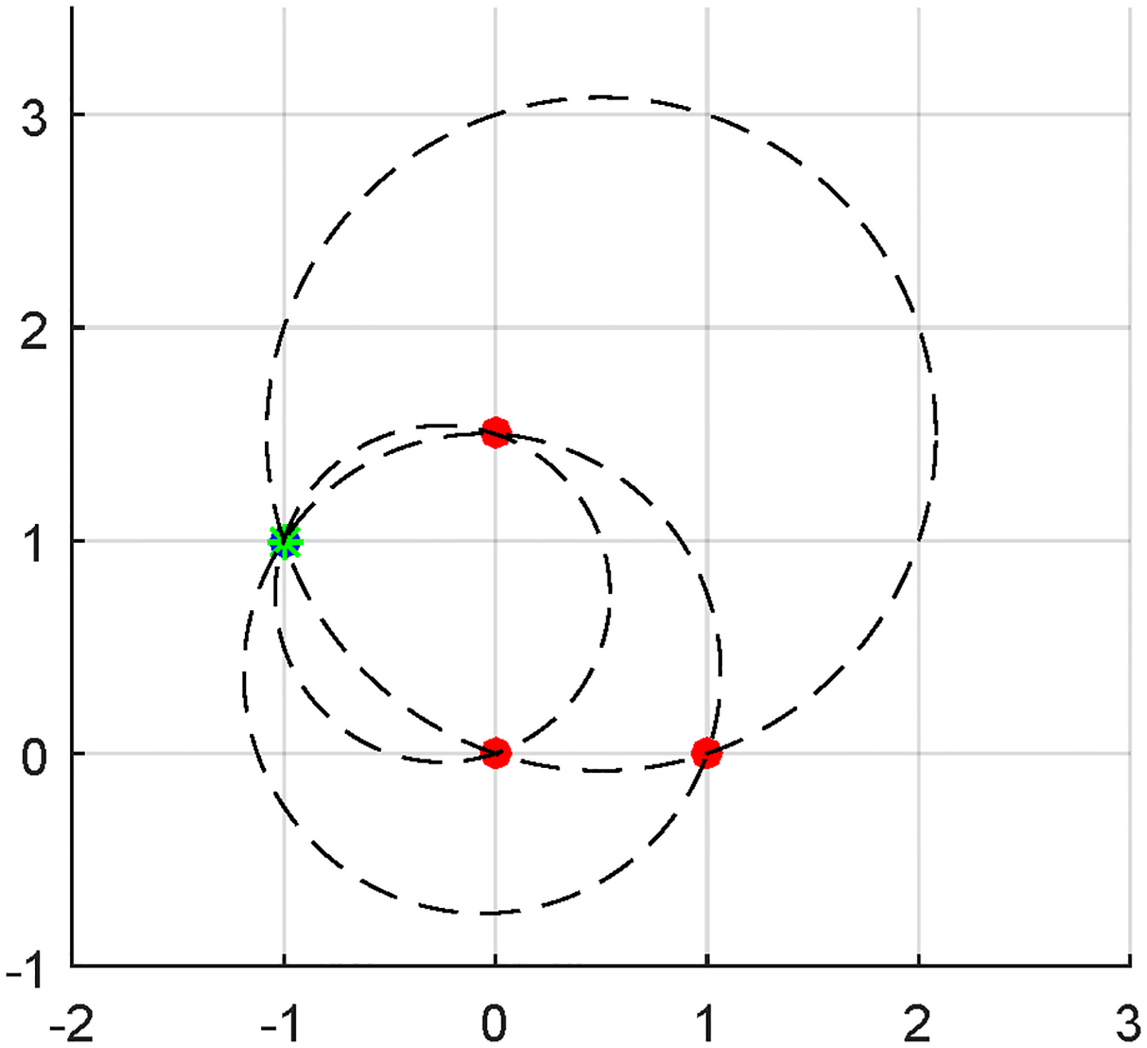
Correct location of a tracked object using three beacons. The beacons are indicated by red circles. The set of plausible curves used for location determination are shown as dashed curves, and the object location at the unique intersection of the plausible curves is indicated by a green star.

To verify the correctness of the proposed algorithm, we provide simulations in which the ground truth of the object location and angle relative to the horizon are preselected. Then the beacons’ locations and the inner products of angle of incidence from pairs of beacon signals are available to perform computations, as would be the case for an on-board antenna array with a microprocessor. By following the algorithm explained in the previous section, it is sometimes possible to exactly recover the true object position with only three beacons and no other information. A diagram of such an example is presented in Fig 2, where only one feasible intersection point exists. This intersection point must be the location of the tracked object.

**Fig 3:**
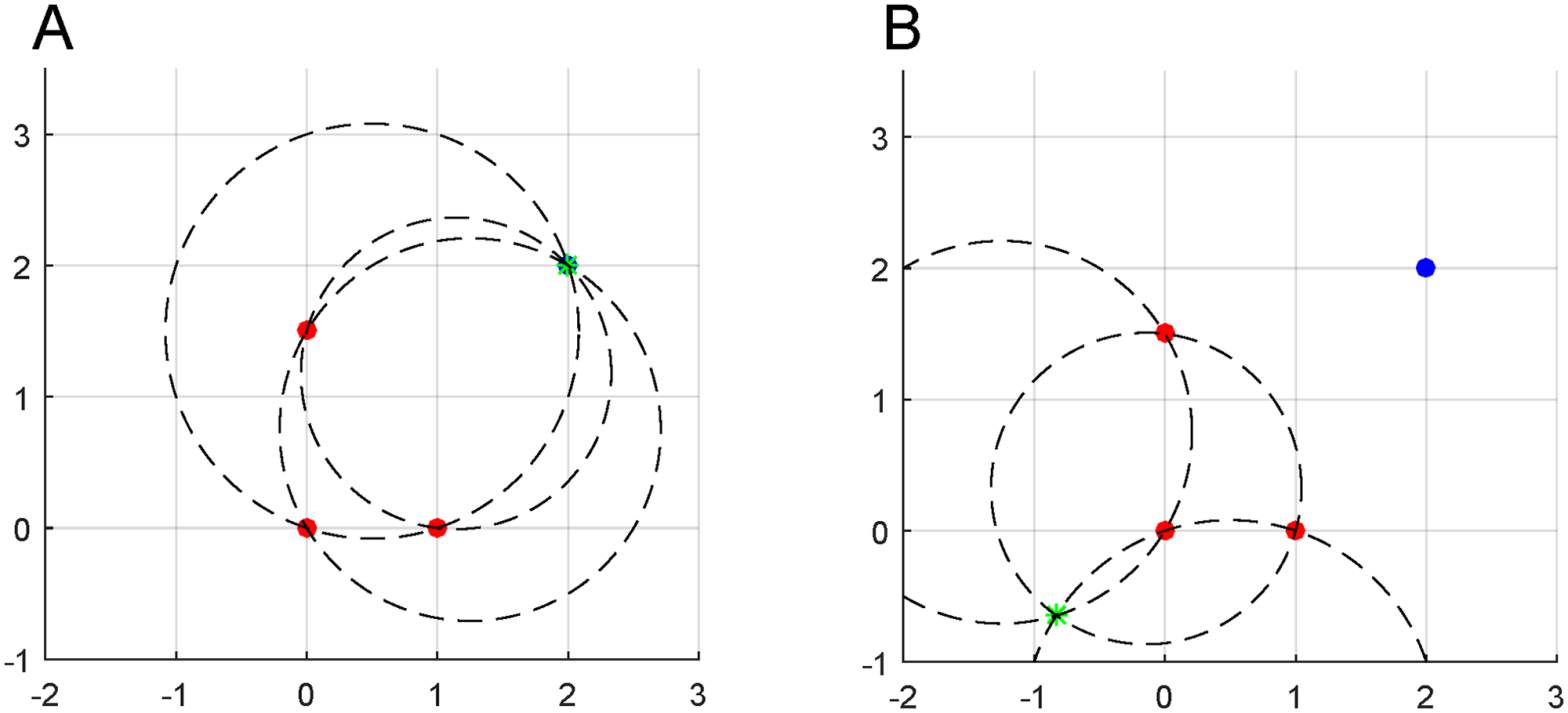
Multiple predicted locations of a tracked object. Here, three beacons (red circles) are used to perform on-board triangulation of the true location of the tracked object (blue circle). The dashed circles denote plausible curves of each pair of beacons which were used to determine the marked intersection point (green star) correctly in A and incorrectly in B.

However, there are also cases where three beacons is not sufficient to uniquely determine the location of the object using the angles computed by on-board antenna arrays. This is because, in some cases, a 180° degree rotation of the tracked object at the true location would be indistinguishable from the original orientation but at a different location. An example of this is seen in Fig 3, where two feasible intersection points exist and only one is the true object location. Therefore, in such cases, additional information is required to determine which predicted position is correct. Such additional information could include knowledge of the half space in which the object lies, a recent past location of the object for a proximity determination, or the signal from a fourth beacon. In particular, if half-space information is known in the application at hand, a directive antenna can be used for one of the beacons to blind the antenna (and hence the algorithm) to the false location.

**Fig 4:**
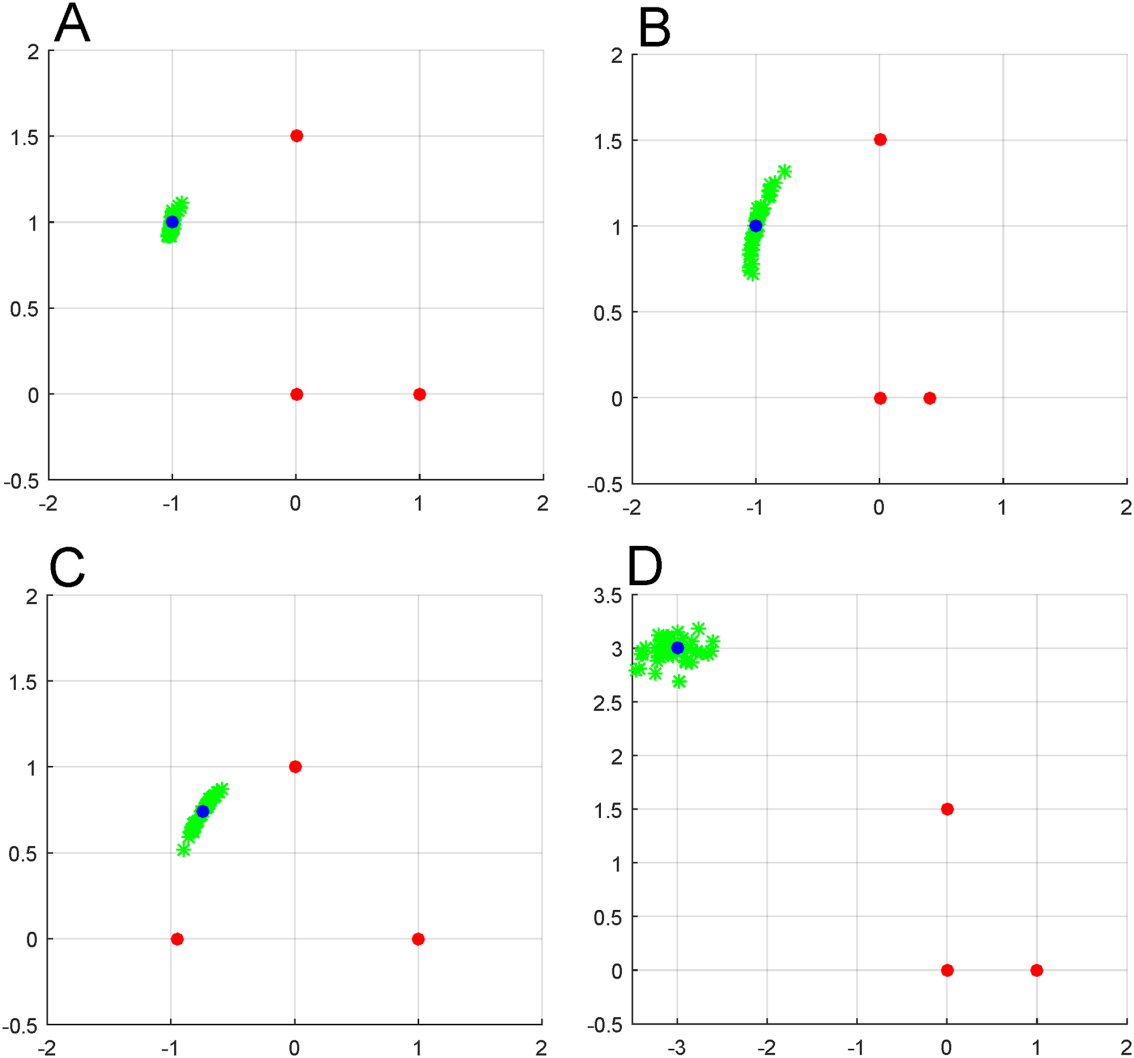
Locating a tracked object under noisy measurements. The beacons are again noted by red dots, the true location of the object by the blue dot, and the computed locations under noise are shown as green asterisks.

Additionally, our discussion has been of the idealized condition in which measurement noise is neglected. While noise can be reduced by precise measurements and long signal integration times, in any implementation of this algorithm, some noise is inevitable and should be taken into account. Simulations in which the information reaching the tracked object is corrupted can be performed to compute the accuracy in different scenarios. Depending on the configuration and number of beacons, the level of noise sensitivity will vary. We provide some examples in Fig 4 in which on-board triangulation is performed using three beacons and the on-board angle measurements are corrupted with Gaussian noise of standard deviation 0.5° to demonstrate the error this will induce in object position predictions. (The configuration of beacons is diagrammed as in Fig 2 and uses the same symbols). This figure demonstrates that the predictions are relatively robust to noise. However, because the noise sensitivity can lead to poor performance under certain configurations of the beacons relative to each other and the target, we highlight two of the primary sources of such error. In (Fig 4B and 4D), the angle between beacons from the perspective of the object is small, so that fluctuations cause the corresponding plausible curves to fluctuate more heavily with noise. A second source of sensitivity is when the object lies near the circle that the beacons lie on as in Fig 4C. When the beacons and object are exactly on the same circle it becomes impossible to locate the object on that circle, so it is not surprising that the algorithm breaks down near that scenario, when the distance *d* between the centers of intersecting plausible curves is low.

The sensitivity can obviously be reduced in an implementation of this algorithm by including historical information, integration of signals to reduce noise, the use of additional beacons or even moving the beacons further away from one another. A more general extension of this work would be to increase the number of beacons and use on-board estimation of the sensitivity of different sets of beacons to weight location predictions of subsets of the beacons based on their robustness to improve accuracy.

## Discussion

In this letter, we propose a variant on standard triangulation which would be performed entirely using on-board measurements and can accurately reconstruct the location of a tracked object. The algorithm could be implemented using dumb beacons placed at locations known to the tracked object, and antenna arrays on the object to determine the direction of these incoming signals. We have described a calculation rooted in classical geometric theorems which is distinct from that of standard triangulation, and has the advantage of autonomy: The location of the object is never broadcast to it. Numerical results also showed that the computation is reasonably insensitive to noise.

There are several future directions and extensions which would be helpful for implementing this idea. Three-dimensional tracking is an obvious extension which complicates the mathematical implementation but does not alter the essential geometric intuition underlying the algorithm. In 3D, the minimum number of beacons changes from three to four, and the plausible curves change from circular arcs to rotations of circular arcs about a chord. The analytic solution described here for the intersection of plausible curves does not apply, so that a numerical method for finding the intersection of the surfaces is required. In addition, further study of the effects of noise and beacon configurations would also be of interest. On-board angle detection via smart antenna arrays has been proposed to detect and mitigate spoofing [13–15], and it would be an interesting direction to consider spoofing attacks on the positioning system described in this work. Finally, a more realistic treatment of the electromagnetic environment might help to clarify what sort of technology would be best for the differentiation of beacons.

## Supporting information

S1 File The simulated noisy data used to generate Fig 4A. (XLSX)

S2 File The simulated noisy data used to generate Fig 4B. (XLSX)

S3 File The simulated noisy data used to generate Fig 4C. (XLSX)

S4 File The simulated noisy data used to generate Fig 4D. (XLSX)
